# Breaking barriers, changing paradigms: Africa's radical agenda for HIV sustainability

**DOI:** 10.3389/frph.2025.1612902

**Published:** 2025-08-07

**Authors:** Mumbi Chola, Magda Robalo, Kent Buse, Pokuaa Oduro-Bonsrah, Jekwu Ozoemene, Abdoul Dieng, Ruth Akulu, Bernard Madzima, Awa Marie Coll-Seck, Robb Sheneberger, Sesupo Makakole Nene, Izukanji Sikazwe, Michel Sidibe

**Affiliations:** ^1^ Adult HIV Department, Centre for Infectious Disease Research in Zambia, Lusaka, Zambia; ^2^ Institute for Global Health and Development (IGHD), Bissau, Guinea-Bissau; ^3^ Global Health 50/50, Cambridge, United Kingdom; ^4^ HIV Trust Fund of Nigeria, Lagos, Nigeria; ^5^ Nigeria Business Coalition against AIDS (NiBUCAA), Lagos, Nigeria; ^6^ AD Consultant, Abidjan, Cote d’Ivoire; ^7^ ACTS101, Kampala, Uganda; ^8^ Zimbabwe National AIDS Council, Harare, Zimbabwe; ^9^ Galien Forum Africa, Dakar, Senegal; ^10^ Centre for Infectious Disease Research in Zambia, Lusaka, Zambia; ^11^ SCMN Global Health Consulting, Pretoria, South Africa; ^12^ Africa Medicines Agency, Bamako, Mali

**Keywords:** HIV response, leadership, sustainability, Ubuntu, Africa

## Abstract

Despite significant progress in the HIV response, the sustainability of this journey is threatened by over-reliance on external support and imported and often inappropriate models. The recent sudden shifts in the United States Government's foreign aid policy have heightened the urgency for independence. Africa is at a critical point, which presents an opportunity to move from dependency on external assistance to establishing itself as a self-sustaining center of innovation and sustainable growth. Africa must reshape its approach to the HIV response by addressing the continent's over-reliance on external funding and shift towards self-sustainability and inclusiveness. For Africa to sustain its HIV response, it is critical to have African voices and leadership in the HIV response, adopt African-centric approaches in moving from silos to the integration of programme governance, ensure renewed governance and accountability frameworks, Africanizing research and development and also ensure African medicines security and sovereignty. Africa must leverage Ubuntu approaches to empowering communities, women, youth, and key and vulnerable populations, and work with community networks for service delivery. There must also be sustained HIV Programmes in Fragile and Post-conflict Settings. It is also critical to secure domestic financing through a continental approach to financing health and well-being. For Africa to realize the vision of a sustainable, African-led, and owned HIV response and health agenda, collective action is imperative. African stakeholders must fully support this agenda and claim it as their own in the spirit of Ubuntu, within the context of continental plans for transformation and revitalization. Together, we can realize the vision of the “Africa we want.”

## 1 Introduction

### 1.1 A critical juncture

Donor countries in the global north, including the United States, Germany, the Netherlands, Britain, and France, recently cut funding towards development assistance and the global HIV response (1, 2). These funding cuts have negatively impacted Africa's HIV response (3). As of 2023, 65% of people living with HIV globally were in sub-Saharan Africa and have been significantly impacted by these funding cuts (4). Amidst the current shifts in the development assistance for health and the global health landscape, Africa must transition from dependency on external assistance to establishing itself as a self-sustaining center of innovation and sustainable growth. Although the majority of African countries are heavily reliant on donor support due to various economic challenges such as high debt burden, limited domestic resource mobilization, donor-driven agendas and policy conditionalities, vulnerability to external shocks, structural weakness in public financial management, and historical and structural legacies such as colonialism (5, 6), this transition is necessary for the sustainability of the health programs and the HIV response.

This transition must be rooted in the philosophy of Ubuntu, which underscores the essence of mutual support and interconnectedness. With its abundant human and natural resources and a dynamic, youthful population ready to reshape its story, Africa must harness these assets not only to meet health challenges, such as HIV and non-communicable diseases, but also to secure political, cultural, and economic autonomy. This paradigm shift aligns with the African Union's Agenda 2063, “THE AFRICA WE WANT” (7), which advocates for self-reliance, prosperity, and well-being for everyone on the continent. It marks an era where the continent not only has a seat at the global table but also owns it and relocates the meeting table to Africa, directing its journey towards sustainable economic development for all.

Scientific advances and best practices in diagnostic, care and treatment tools and interventions have led to the adoption of innovative technologies, propelling more than forty countries globally in 2021 to be on track to end AIDS as a public health threat by 2030 (8). African countries have made significant strides in combating HIV and AIDS, resulting in expanded access to lifesaving antiretroviral therapy, reduction in AIDS-related deaths and huge declines in annual new HIV infections in particularly in eastern and southern Africa (57% reduction) and western and central Africa (49% reduction) between 2010 and 2022 (9). In 2022, countries including Botswana, Eswatini, Rwanda, Tanzania, and Zimbabwe achieved or exceeded the Joint United Nations Programme on HIV and AIDS (UNAIDS) 95-95-95 targets (9), with 16 more countries, including eight from sub-Saharan Africa, on the brink of reaching these targets (9) sparking optimism about the possibility of ending AIDS as a public health threat by 2030. Effective HIV treatment has significantly reduced global AIDS-related deaths by 52%, from 1.3 million in 2010 to 630,000 in 2022. In 2023, 93% of people living with HIV (PLHIV) in Eastern and Southern Africa (ESA) knew their HIV status, 83% of people living with HIV received antiretroviral therapy, and 78% of PLHIV achieved HIV viral suppression, while in Western and Central Africa (WCA), the cascade was 81%-76%-70% (8, 9).

However, even with this progress, regional, sub-regional, and sub-population disparities persist. Data from 2023 shows that new HIV infections are also rising in several African regions and countries, with significantly more new infections being recorded in ESA and WCA among children (50,000 vs. 48,000), adolescents, and young people (160,000 vs. 49,000), and adults (400,000 vs. 140,000) (10). Disparities also persist in the Prevention of Mother-to-Child Transmission (PMTCT) and Elimination of Mother-to-Child Transmission (EMTCT) across Africa, reflecting regional variations in healthcare access, HIV prevalence, and program effectiveness (11). Two-thirds (68%) of HIV infections in children in WCA were due to mothers not receiving antiretroviral therapy, while about one-third (32%) of new infections in children in ESA were due to their mothers' inability to avoid acquiring HIV during pregnancy or breastfeeding (11). There were also disparities in the testing and treatment cascade with WCA and MENA regions well below the 95-95-95 targets compared to ESA. Data shows that children are lagging behind adults across the treatment cascade (10).

At this critical juncture, where HIV infections among young people are on the rise and declines in mortality rates are sluggish (11), involving key and vulnerable populations becomes even more pivotal. These African citizens, facing marginalization and criminalization, are instrumental in altering the course of the HIV epidemic. This scenario underscores a looming crisis, highlighted by the acute risk to 190,000 untreated pregnant women in 2023 (11). The rise in new HIV infections in North African countries such as Algeria, Egypt, and Sudan is also a cause for concern (12). Immediate, comprehensive interventions are imperative to bridge service gaps and reinforce prevention and treatment mechanisms, aiming to mitigate the epidemic's potential escalation.

The HIV response on the continent has been largely externally funded, with the United States President's Emergency Plan for AIDS Relief (PEPFAR), the Global Fund, with PEPFAR alone investing over $120 billion since 2004 (13). The over-reliance on external support and imported, often inappropriate models (14), particularly financial resources, coupled with underinvestment from African countries, threatens to jeopardize this progress. The failure to meet the Abuja Declaration's funding goals (15), illustrates a gap that endangers the sustainability of our health progress and victories. Funding towards HIV has been declining in recent years, further exacerbated by funding cuts from the United States Government (USG) and European countries such as the UK, France, Germany, and the Netherlands (16, 17). The effects of the Executive Orders from the White House on the re-evaluation and realignment of the USG foreign aid (2), included the disruption of prevention and treatment services, especially for key populations and adolescent programmes, human resource shortages, and challenges with access to patient data (4). This showed the negative effects of dependence on external resources and emphasizes the need for Africa to look inwards for lasting solutions. Although partnerships with external funders remain critical, it is crucial for Africa to have a well-designed and articulated transition plan as it advances towards shifting away from this health dependence paradigm (5).

The transition towards self-reliance and driving the HIV response requires bold African leadership. There is an urgent need for renewed African leadership and enhanced forms of inclusive governance modelled on Ubuntu principles (18), from the grassroots right through to the continental level, cannot be overstated. These collaborative and inclusive strategies must prioritize the health and dignity of the most vulnerable groups, focusing on both prevention and treatment. This paper outlines a vision for an African-led sustainable HIV response and the critical components required to achieve it.

### 1.2 Vision

This moment calls for “Breaking Barriers, Changing Paradigms”, a vision that encapsulates Africa's determination to decolonize the HIV response and achieve improved governance and financial independence and deepen community engagement in ending AIDS. Overcoming the range of challenges ahead requires a collective, unified effort imbued with hope and determination in Africa's potential to shape its own future.

Africa's pursuit of sustainable HIV responses necessitates more than just a reset of its financing. It demands a holistic review of strategies, policies, and cultural norms while tapping into latent opportunities. It is essential to dismantle systemic barriers, address ingrained biases, and ignite grassroots movements for lasting change.

“Breaking Barriers, Changing Paradigms” is not just a slogan; it is a rallying cry for a transformative shift that extends beyond the sustainability of our HIV responses. It is a cry that was born from the African-led HIV Control Working Group (HCWG), a think tank comprised of 12 African experts with diverse and extensive experience in HIV programme implementation, policy, advocacy, and research, with the aim of creating thought and dialogue about African health and HIV sovereignty (https://hivcwg.org/). It aims to cultivate a fairer, more equitable, and resilient society. Living through colonization creates a new appreciation for the word “empowerment” when forced disempowerment has been the experience, which those outside Africa may fail to appreciate. It envisions empowering every African to live AIDS-free with reduced risk of new HIV infections- meaning the options to choose treatments, prevention, and human right protections like persons in the global north largely enjoy, and where, like in the global north, health and well-being are a universal birthright and not privileges.

This vision is a long-term one that requires the same courage and determination required to gain independence from colonial mindsets, to advance to the next step of Africa's journey to create a sustainable HIV response. It transcends borders, challenges norms, and requires shifts in the mindsets of African leadership and its people to lay the foundation for a brighter, healthier future for all people living on the continent.

The road to achieve the vision begins with the recognition that deconstructing a colonized approach to African development will likely take decades, requires broader lenses than just the HIV response, and must be done in collaboration with the global community. This paper outlines what Africa must do to achieve this.

## 2 Critical components of a radical agenda for HIV sustainability

UNAIDS defines HIV response sustainability as “a country's ability to have and use, in an enabling environment, people-centered systems for health and equity, empowered and capable institutions and community-led organizations, and adequate, equitably distributed, resources to reach and sustain the end of AIDS as a public health threat by 2030 and beyond, upholding the right to health for all.” (19) Sustaining the HIV response will need to be nuanced and fit for purpose, depending on country and regional context, considering the heterogeneity of the epidemic. This may also require countries and regional bodies to contextualize their definition of HIV sustainability. To contextualize the sustainability of the HIV response in the African context, we recommend the following critical components that should be included.

### 2.1 African voices and leadership in the HIV response

#### 2.1.1 Amplified African voices in shaping the HIV response

The HIV response has been successful in expanding access to life-saving treatment due to global solidarity, strong political leadership, inclusive governance, and accountability. However, the global governance structure is biased towards the global north, despite Africa bearing the largest burden of HIV (20), with the global north, through donor programs such as PEPFAR and The Global Fund, and institutions such as USAID, the Bill and Melinda Gates Foundation, setting and driving the agenda. Even in institutions such as UNAIDS and the World Health Organization (WHO) that have inputs from countries, the agenda is largely driven by countries in the global north who contribute large amounts to these institutions. To amplify African voices in global health decision-making, it is crucial to strengthen African leadership in the HIV response on the continent in order to influence the global HIV response. With the understanding of the context and nuances that influence the success of interventions on the continent, Africa must shape its HIV response to ensure its success and sustainability. Furthermore, to align the global HIV response with the nuances of the African HIV response, it is important to increase African representation in decision-making positions globally. This will align global health leadership with local and African contexts, advocating for evidence-informed policies that reflect the continent's diverse experiences, challenges, and needs. A unified African stance in global health narratives is essential. To have a truly strong and influential African voice in the HIV response, it is vital to have African leadership represented in governance structures that shape the HIV agenda at all levels, from global, regional, local, to community levels. The lived experiences and local contexts of people affected by HIV, their communities, and civil society must be central to governance structures, and their meaningful participation in global and national decision-making forums. This involvement is crucial for strong political support and improved accountability of governments, implementers, and funders in meeting financial commitments and delivering more equitable and effective services (21).

### 2.2 African-centric approaches in moving from silos to the integration of programme governance

#### 2.2.1 From vertical silos to strengthening of integrated government thinking and systems

As we transition to sustainable HIV programming, it is important to have an efficient and well-coordinated HIV response. Global health initiatives in HIV have been operating vertically, often bypassing country systems and disrupting efforts to strengthen health systems and coordinate disease programmes, resulting in the re-verticalization of planning, management, and monitoring and evaluation systems (22). Misplaced logic and inappropriate models often lead to issues in addressing local contexts, effective use of funds, stakeholder involvement, and performance and outcomes (23). Therefore, African governments must transition towards the integration of their health systems. Integration of HIV services into primary healthcare has been identified as a key step towards sustainable HIV control (24, 25). It is considered a key pathway towards achieving universal health coverage (26). Integration will likely involve developing locally relevant and contextual models for integration into primary healthcare, across the entire system, and the health system building blocks. Through integration, African governments can address challenges posed by verticalization by streamlining the parallel and duplicative processes from the different initiatives, improving country donor coordination processes, and the general budget support (GBS) mechanisms (27–29).

### 2.3 Renewed governance and accountability frameworks

#### 2.3.1 Mutual accountability

Just as donors expect transparency and greater accountability from the recipients regarding how resources are allocated and utilized, recipients of these resources also demand transparency and greater accountability from society, donors, and other development partners (30). This particularly refers to institutions, on both sides, involved in governance, implementation, and management of the localized HIV response, with specific regard to their ﬁnancial and non-ﬁnancial affairs. Transparency in the allocation of funds and programme actions is essential to gain widespread support from all stakeholders involved in addressing the looming funding crisis for HIV programming in Africa (30). Transparency is also important in how recipients use the funds to ensure they are used for the intended purpose. Mutual accountability between donors and African governments, and governments with the broader community, is vital to the transparent and efficient use of resources allocated to the HIV response (31). It fosters a balanced partnership between donors and recipient governments by binding members together through shared values and reciprocal commitments in a voluntary process (31). This involves ensuring there is fair representation in decision-making on fund investments, with transparency and independent monitoring of funding mechanisms and allocation, and the use of resources. Furthermore, to inspire donor confidence in how African governments utilize donor funds, they must also address the widespread corruption and mismanagement of funds by instituting robust fiscal management and monitoring systems.

#### 2.3.2 Community monitoring

A component of mutual accountability, particularly within countries, the role of community-led monitoring between governments, the communities they serve, and civil society organizations (CSOs) is essential. Different models of such monitoring have been established with varying degrees of success in some countries (32). Community-led monitoring has helped improve health outcomes such as better maternal and child health, increased immunization rates, and enhanced health infrastructure, including building renovations and equipment. It has also contributed to improvements in healthcare provider behavior, such as more responsiveness, friendlier attitudes, reduced tardiness, and absenteeism from health workers (32). There are, however, documented challenges faced by community-led monitoring structures, such as financial resources and technical expertise, which are among the vital requirements for enabling and capacitating communities with knowledge of the key issues relevant to, and processes for, community monitoring (32). Having communities capacitated to fully participate–as stakeholders and partners rather than tools for data collection–in monitoring key indicators of the progress of the HIV response will help hold governments and programme implementers accountable. At the continental level, the African Peer Review Mechanism (33, 34) is a powerful tool that countries can use to benchmark progress and monitor their progress towards achieving HIV control and sustaining it.

#### 2.3.3 Putting a sustainable HIV response on the agendas of regional political and economic bodies

Adopting a regional approach will be crucial for the sustainability of the HIV response, considering the region-specific profiles of the HIV epidemic in Africa, which require region-specific responses. Regional organizations are collectively “owned” by member states and other regional actors and therefore present opportunities to raise the HIV agenda and its governance to the forefront of agendas. This could aid in driving compliance among member states to meet agreed benchmarks (21) by galvanizing actions among member states and introducing more harmonized approaches, including services for populations in border locations, and pooled production and distribution and medicines.

### 2.4 Africanizing research and development

African health research efforts, including HIV research, have been externally driven, and financially supported, both at the organizational level and at the level of individual scientists (35, 36). This approach erodes efforts for sustainable production of African-led knowledge. Between 2010 and 2017, researchers from Africa and the Middle East participated in only 21.6% of the global scientific research on HIV and AIDS (37) while between 2019 and 2021, only 17.9% of Africans appeared as first authors on randomized control trials on HIV (38). Despite this low participation, Africans remain best placed to identify and contextualize the most locally relevant and pressing problems. Research led by African scientists is more likely to address locally relevant problems and is more likely to be communicated in a cultural and policy context that is more accessible and relevant to local populations and decision-makers (35, 39, 40). The significant increase in research and capacity-building investment in recent years on the African continent has been encouraging (41, 42). Although these investments have come primarily from the global North, many African governments are increasingly providing funding towards local and regional scientific research and capacity-building (42). Overwhelmingly, calls to increase local funding towards research and development have arisen across the continent to effectively address research questions that are a priority in Africa. South-to-south collaboration among African countries presents a strategic approach for building sustainable research hubs of excellence that will leverage expertise from within the continent while harnessing and integrating indigenous knowledge to tackle problems within local contexts.

#### 2.4.1 Securing African data (including research, clinical trials, and personal health records)

Due to Africa's disproportionate HIV burden, a significant amount of innovative research has taken place on the continent. However, African governments and research institutions struggle with leading the formulation of the research agenda, ownership, and control of the data generated in Africa, due in part to limited local investment. Northern dominance and entitlement among the researchers and funding bodies, inadequate attention to principles of fair research partnerships, and poor negotiation by Africans also contribute to Africans' lack of leadership in research. African investigators comprised of less than one-third of global HIV scientists serving as principal investigators of 4,823 studies in 2020 and 2021, and similarly, only 23% of HIV research has been led by a global south institution (37, 43). This has also compromised ownership of data generated from this research, as funding agencies insist on ownership of the data.

In this data-driven world, African leaders must ensure that health research data generated in Africa is owned and controlled by Africans and used to address health challenges on the continent. African leaders, research and development partners, and regulatory bodies must actively support data sovereignty. By doing so, Africa can bridge the research-to-implementation divide, investing in research on the continent and accelerating the adoption of leading treatment and prevention interventions. Data sovereignty is crucial for empowering African countries to invest in and accelerate health interventions. By ensuring local control, enhancing security, and fostering trust, data sovereignty can lead to more effective, context-specific, and sustainable health outcomes. Strengthening data governance and infrastructure is essential for leveraging the full potential of health data in addressing the continent's health challenges.

### 2.5 African medicines security and sovereignty

#### 2.5.1 Continental regulatory harmonization

Africa has the potential to produce drugs and medical commodities locally at scale. Significant investments are needed to ensure local production meets the demand on the continent. There are key enablers that can help Africa realize this vision, which include political commitment and thought leadership at the top level, coordination between governments and different regulatory authorities, and strong regulations to ensure high-quality products. Effective drug pricing mechanisms to determine the cost of pharmaceutical products are essential to ensure equitable access to essential medications, incentivize innovation in pharmaceutical research and development, and maintain healthcare system sustainability. The African Medicines Agency (AMA), a specialized health agency of the AU, which builds on the Africa Union Development Agency—the New Partnership for Africa's Development (AUDA-NEPAD)'s African Medicines Regulatory Harmonization (AMRH) initiative (2009), will play a crucial role in strengthening the weak regulatory systems and leverage the African Continental Free Trade Area (AfCFTA) to market medical commodities and products (44, 45).

#### 2.5.2 Local capacity

It is also vital to develop local capacity to produce these commodities, promote pooled procurement to create a demand-driven market, protect local manufacturers and markets, and promote competition among manufacturers to encourage innovation (46).

There are, however, various challenges that can impede this endeavor, which include difficulty with obtaining prequalification approvals from the World Health Organization (WHO) and U.S. Food and Drug Administration (FDA), lack of guaranteed market for products, high import duties and taxes, financial constraints, and insufficient government commitment (47). Africa also has a variety of regulatory requirements for pharmaceutical production that vary across regions and countries. Furthermore, the local manufacturing industry of active pharmaceutical ingredients (API), a key ingredient in the production of pharmaceuticals, is not well established in Africa (48). Local manufacturers face a cost disadvantage because while they can import finished medicines duty-free, they must pay duty on APIs, which account for 60% or more of the final cost of the product.

Key to local pharmaceutical production is the harmonization of the regulatory requirements for pharmaceutical production which vary across countries and regional bodies such as the East African Community (EAC), Southern African Development Community (SADC), Economic Community of Central African States (ECCAS), Economic Community of West African States (ECOWAS), Arab Maghreb Union (UMA) and Community of Sahel–Saharan States (CEN–SAD). Continental regulatory requirements are essential for promoting a conducive environment for pharmaceutical production in Africa. They play a critical role in ensuring the availability, quality, and safety of medicines, supporting economic development, and improving public health outcomes across the continent.

#### 2.5.3 Political commitment to African medical security and sovereignty

Africa bears a quarter of the global burden of disease (41) and imports between 70% to 97% of its pharmaceutical commodities (46, 49–51). African drug manufacturers often import machinery, packaging, and active pharmaceutical ingredients (APIs) due to weak local production capacity. The COVID-19 pandemic exposed the risks of such overdependence when disruptions in the global supply chains and countries restricting the export of medicines were experienced. This has fueled the push towards Africa producing its commodities locally. However, ensuring reliable and sustainable manufacturing of medicines and other health technologies is not only complex but will require highly accountable and strategic partnerships (52).

Political commitment, long-term financing, and a clear vision, along with supportive national policies, good governance and rule of law, robust national regulatory authorities and other relevant institutions, diverse technical expertise, and access to viable markets are all essential to the success of local production in Africa (52). Policies such as direct fiscal and non-fiscal incentives by governments to local manufacturers, review of tax burdens and incentives, supporting pharmaceutical industries to attain WHO prequalification, strategic joint ventures with international pharmaceutical companies, and multisectoral collaboration are all important for success (47). The establishment of regional hubs for leveraging expertise and creating economies of scale is also critical to securing African sovereignty in this space (50).

#### 2.5.4 Pooled procurement

This is also an option for African countries to manage cost, improve access and efficiency of procurement of medicines, vaccines, and medical commodities, and leverage limited technical capacity and human resources incentives to manufacture or supply specific medicines or vaccines (53, 54). Pooled procurement has been promoted to address challenges related to limited access to affordable, quality-assured medicines, small market size of the buyer, limited technical capacity and human resources, and insufficient incentives to manufacture or supply specific medicines or vaccines (53). African countries must invest in establishing mechanisms that can ensure the success of pooled procurement. For example, since 2005, the East African Community (EAC) has made limited progress in implementing an inter-country pooled procurement mechanism. The lack of dedicated funding and ownership to drive the project forward led to the stagnation of the process for some years (55). To successfully implement pooled procurement, African countries must continue to align efforts, sustain political will, and allocate sustainable funding towards pooled procurement (55).

### 2.6 Leveraging Ubuntu approaches to empowering communities, women, and key and vulnerable populations

#### 2.6.1 Ubuntu

Ubuntu serves as the spiritual foundation of many African communities and cultures and represents core values such as respect for human dignity and human life, collective sharedness, humility, solidarity, caring, hospitality, interdependence, and communalism (56). The concept of Ubuntu has been applied in numerous interventions to fight HIV and AIDS (57–59). Historically, it has been at the core of community interventions and has been instrumental in addressing stigma and discrimination (57). In reshaping the African HIV response, the approach towards achieving HIV control and sustaining it must be enshrined in the philosophy of Ubuntu. With a foundation in Ubuntu, as detailed in Box 1, the HIV response must be based on core values, including social justice and dignity for all, transparency and mutual accountability, interconnected health and wellbeing, respect for cultural diversity, inclusivity and equity, community empowerment, decolonizing global health architecture, and social and economic rights for all. These principles are in line with the African Union's Agenda 2063, which seeks to build “The Africa we want” (7) and we call for their realization in our African agenda for AIDS sustainability

BOX 1 **Embracing Ubuntu: a path to collective health, resilience, and sustainable responses to HIV.**

**Table:** 

The ethos of Ubuntu offers a profound perspective for Africa's path towards innovation and sustainable development. It champions a development model where success is gauged not by individual achievements but by the upliftment of the community. This collective endeavor towards development is pivotal in navigating the continent's response to HIV and ensuring no one is left behind in this pursuit.
Adopting Ubuntu in the HIV response heralds a transformative pathway, emphasizing community-led initiatives and an inclusive health model that views the fight against HIV as a communal challenge. It underscores the significance of fostering a sense of unity and shared destiny, thus nurturing resilient, supportive communities where individuals living with HIV can lead dignified, healthy lives. Through this collective effort, Africa strengthens its resolve to overcome one of its most significant health challenges, reinforcing the interconnectedness and mutual dependency of its people. This is Africa's march towards building a sovereign and sustainable paradigm for addressing the HIV epidemic, guided by the principles of Ubuntu, financial autonomy, and innovation.
“**I am because we are.”—Ubuntu Philosophy**

#### 2.6.2 Protecting the rights of the vulnerable

Communities are central to sustainable HIV responses through their participation in prevention, promotion, and care as well as their efforts in community development (60). However, various groups in these communities remain at increased risk of HIV. These include adolescents and young people, women and girls, key populations, mobile populations, prison and incarcerated populations, orphans, and vulnerable children, as well as people living in high-prevalence areas (61–63). In many African countries, some of these groups are marginalized, criminalized, or in conflict with the criminal justice system, making it difficult for them to access health services for fear of arrest and prosecution (64).

To ensure access and utilization of HIV services free of stigma and discrimination, whether structural or societal, among these populations, the rights of these community groups must be upheld. Decriminalizing these populations is a key part of ensuring that they can freely access HIV health services. This is particularly important with the evolution of the HIV epidemic, which is increasingly becoming concentrated in these key and vulnerable populations, making them the future of the HIV epidemic in Africa (65).

#### 2.6.3 Gender equity

Women in Africa are disproportionately affected by HIV. Incidence and prevalence are generally higher among women compared to men, especially among adolescents and young adults (9). Various power, socioeconomic, demographic, and risk behavior-related factors contribute to the higher incidence and prevalence of HIV among women (66). Higher rates of illiteracy, unemployment, and impoverishment among women, unequal power relationships, the subordinate position of women relative to men also increase the risk for HIV infection among them (67–70). Therefore, we must be deliberate about dismantling gender inequalities and inequities across the whole continuum of care, including HIV prevention activities. As programmes targeting adolescent girls, young women, and women are being implemented, there must also be deliberate programmes targeting adolescent boys, young men, and men in communities where the health outcomes lag.

For example, men are left behind with higher proportions unaware of their HIV status, lower proportions of ART initiation, more men with WHO stage III/IV AIDS at first clinical visit, and higher rates of 18-month loss to follow-up compared to women (71). With HIV testing and treatment coverage being significantly lower among men, there are more untested, untreated, and off treatment viremic men. This is leading to HIV transmission being disproportionately driven by men (72). As incidence rates decline, most heterosexual transmission of HIV is being driven by men, and their contribution towards onward heterosexual transmission is growing. This is likely due to slower population-level declines in HIV viremia in men (72). As such, men consistently disproportionately contribute to heterosexual HIV transmission than women. Innovative programmes targeting untested and HIV-positive untreated men for HIV testing, ART initiation, and retention to ensure viral load suppression are critical to curb their contribution to new infections.

### 2.7 Re-shaping health systems

Reshaping health systems for HIV in Africa requires a holistic, innovative, and tailored approach that addresses the unique challenges faced by the continent in ending the HIV epidemic. This will entail addressing challenges in line with the WHO health system building blocks in the context of African norms, values, and unique nuances across sub-regions and countries. Health outcomes can be improved, and transmission rates can be reduced. This can be achieved through expanding HIV testing services and ensuring universal access to antiretroviral therapy (ART) for all those diagnosed with HIV; integration of services by integrating HIV services with other healthcare services, such as maternal and child health, tuberculosis (TB) care, and sexual and reproductive health services, to improve efficiency and reach more people in need. Implementing comprehensive prevention strategies, including promoting condom use, voluntary medical male circumcision, pre-exposure prophylaxis (PrEP), and harm reduction for people who inject drugs, can help reduce new HIV infections. Integration of HIV services across the continuum of care, including prevention services, into primary healthcare is vital for the sustainability of these services and also for strengthening the health system as we leverage best practices from the implementation of HIV.

Embracing and scaling up innovative approaches such as digital health and mobile health (mHealth), telemedicine, and self-testing kits can help reach key populations, improve linkage to care, and support treatment adherence. Strategies such as task shifting and sharing, as well as decentralization through training and empowering non-physician healthcare workers, such as nurses and community health workers, to deliver HIV services, can help overcome human resource shortages and improve access to care, particularly in rural and underserved areas (73). Strengthening health information systems and utilizing data for decision-making can help track progress, identify gaps, and allocate resources effectively. It is also important to improve efficiency in HIV programming to ensure more prudent use of resources as well as reduce the cost of implementing these programmes. Integration of HIV services and utilizing multi-sectoral approaches could help achieve this.

Holistic approaches that address underlying socioeconomic factors such as poverty, gender inequality, and lack of education are crucial for addressing the root causes of the HIV epidemic and improving health outcomes. Furthermore, ensuring supportive policy and legal environments that address criminalization and discrimination against key populations is vital for increased access to HIV services.

A critical component of this is universal health coverage (UHC). UHC is critically important for Africa for several reasons, ranging from improving health outcomes to promoting economic growth and social stability. UHC offers improved health outcomes through improved access to health services, financial protection to citizens by reducing out-of-pocket expenditure, equity, and social justice through equal access and addressing health disparities across different population groups. UHC also has economic benefits by contributing to a healthier workforce and poverty reduction by preventing health-related financial crises. UHC contributes to health system strengthening by encouraging the development of comprehensive and cohesive health systems. This includes having the necessary infrastructure, training health professionals, and supply chains for medicines and equipment. Additionally, UHC drives improvements in the efficiency and quality of health services through better planning, resource allocation, and monitoring. UHC is vital for Africa's development, providing numerous benefits that extend beyond health to economic growth, social equity, and resilience. Investing in UHC can create healthier, more equitable, and more prosperous societies, making it a cornerstone for sustainable development in the region. An example is the Community-based Health Insurance (CBHI) in Rwanda (74). By implementing these strategies within a comprehensive and integrated framework, health systems in Africa can be reinvigorated to effectively address the HIV epidemic and work towards achieving the goal of ending AIDS as a public health threat by 2030. By implementing these strategies within a comprehensive and integrated framework, health systems in Africa can be reinvigorated to effectively address the HIV epidemic and work towards achieving the goal of ending AIDS as a public health threat by 2030.

### 2.8 Sustained HIV programmes in fragile and post-conflict settings

#### 2.8.1 Integration of sustainability into the emergency preparedness and response plans

The COVID-19 pandemic caused chaos and disruption on a global scale. Africa experienced the deadly Ebola epidemic in western Africa between 2014 and 2016, and the cholera outbreak that occurred in southern Africa in 2023–2024. These outbreaks, as well as the COVID-19 pandemic, have led to calls for strong and resilient pandemic prevention, readiness, and response plans, and knowledge from the HIV response infrastructure may be crucial in these efforts. With over 25 million people living with HIV on the continent and more affected by HIV, the HIV response has highlighted the significance of putting human rights and community leadership first, providing important insights for pandemic preparedness and response (PPR). As community and health systems are reshaped and strengthened to ensure they are responsive to future pandemics, lessons and best practices from building the HIV response over the years can be leveraged.

As the HIV response moves toward integrated, person-centered care, especially for women, young people, and people living with HIV, it is critical to address each person's holistic health requirements. Integration must account for the heterogeneity of the epidemic and thus should be tailored and nuanced to respond to the epidemic at the local level.

Furthermore, considering the disruption that the COVID pandemic had on HIV programming, it is important to embed HIV in pandemic preparedness plans to mitigate against similar disruptions. To effectively manage the HIV response and prepare for future pandemics, a people-centered approach must be combined with building robust health and community systems. Leveraging knowledge from Africa's responses to COVID-19 and HIV could be crucial in directing and enhancing international PPR initiatives.

### 2.9 Work with community networks for service delivery

#### 2.9.1 Community health systems and community health workers

Community health workers (CHWs), as a community-based extension of the health system, are essential for the delivery of HIV treatment and care and comprehensive primary health care (75). They are a valuable healthcare cadre that manages and supports the rising number of people who need to be initiated and maintained on ART (76, 77). As communities are enabled to lead the HIV response, CHWs will play a key role in the sustainability of the HIV response. CHW programs, however, face various challenges, including a highly fragmented health system that lacks government ownership and is poorly integrated into national health systems (77). In many instances, CHW programs are donor and implementing partner-led and not government-owned and funded. Enablers for harmonization include recognition of nongovernmental CHW programs, using common incentives and training processes for CHWs, fair compensation and remuneration for CHWs, having an organizational structure dedicated to community health initiatives, and involving community leaders in decision-making (78). It is also important that, as we strive to expand the health workforce, the retention of skilled labor is also emphasized.

The focus of the HIV response is moving towards integrated, people-centered care to address individuals' multiple health needs across life's continuum to support the highest quality of life for people living with HIV. Therefore, it is crucial to build resilient health and community systems, which are tailored to the local context, considering how dynamic the HIV epidemic is across all levels. These systems will help manage the HIV response as well as prepare for future pandemics, deriving lessons from COVID-19 and other lived experiences to ensure uninterrupted access to services.

#### 2.9.2 Socio-political and socio-economic crises

Crises such as armed conflict and forced displacement have affected several parts of Africa with various consequences for the HIV response. Countries and communities affected by humanitarian emergencies have lower coverage of HIV services compared to more stable countries (9). The impact of these crises on HIV incidence and related morbidity and mortality has not been estimated, although effects on the determinants of HIV risk and infection have been documented (79). For example, as of May 2023, 70% of hospitals in Khartoum have closed, resulting in severely disrupted access to HIV services (9). Therefore, the HIV agenda in such African countries affected by humanitarian emergencies must be prioritized within the broader humanitarian response framework. In these settings, evidence-informed, rights-based, and resilient HIV responses must be developed.

### 2.10 A continental approach to financing health and wellbeing

#### 2.10.1 African health and wellness fund

A cornerstone of the future of the HIV response and overall health of the continent is in pioneering innovative and bold financial frameworks at the country and continental levels. Locally developed and driven innovative funding mechanisms will be important for the sustainability of the HIV response in Africa.

At the country level, different domestic financing initiatives for HIV and other health areas have proven successful in generating much-needed local financing. Examples of innovative financing mechanisms or instruments that have proved successful include Zimbabwe's AIDS Trust Fund (a tax/Levy–based instrument), Botswana's National HIV/AIDS Prevention Support (BNAPS) International Bank for Reconstruction and Development (IBRD) Buy–Down (a debt conversion instrument), and Côte d'Ivoire's Debt2Health Debt Swap Agreement (a debt conversion instrument) (80). These instruments have proved successful in raising millions of dollars. Zimbabwe's AIDS Trust Fund generated US$ 52.7 million between 2008 and 2011, Botswana's IBRD Buy–Down generated US$ 20 million, and Côte d'Ivoire's Debt2Health Debt Swap Agreement generated US$ 27 million (80).

At the continental level, akin to global funding mechanisms, Africa needs a funding framework that can be leveraged to finance the HIV response and other diseases, particularly with rapidly declining external funding. A proposed African-owned Health and Wellness Fund is essential as we shift towards the African continent and its countries taking ownership of the HIV response. The African Health and Wellness Fund is a bold statement of Africa's will to control its health destiny. Beyond just being about finances, the Fund is about health resilience, ownership, empowerment, and leading the way in the resurrection of African health. It is how Africa protects its future. This fund, which is run by Africans for people living in Africa, challenges established aid paradigms. It is a daring declaration of sovereignty over our health policies and resources, engaging key players who are sensitive to the pulse of the continent.

In the development of this fund, the private sector is a key stakeholder as they have historically played a crucial role in HIV financing and in ending HIV in Africa. Beyond providing financial support, the private sector provides essential support to complement government and international funding through diversification of funding sources, thus contributing to reducing dependence on external aid and promoting long-term sustainability. Additionally, they also provide innovative financing mechanisms such as Public-Private Partnerships (PPPs) and Corporate Social Responsibility (CSR), efficiency and expertise including operational efficiency, and technology and innovation.

## 3 Conclusion

“Breaking Barriers, Changing Paradigms” is an ambitious call to action, setting out an audacious agenda for HIV sustainability in Africa. Its success hinges on pragmatically addressing the challenges of implementation, ensuring inclusivity, and adapting to the continent's diverse contexts. Its ambitious proposals necessitate a critical examination of feasibility, scalability, and specificity. This narrative underscores the importance of a balanced approach that appreciates our visionary goals while scrutinizing the practicality and impact of its recommendations.

This paper sets forth a transformative agenda aimed at reshaping Africa's approach to HIV response by addressing the continent's over-reliance on external funding and making calls for a shift towards self-sustainability and inclusiveness. The economic and social implications of new domestic funding mechanisms, the political will for implementation, and the potential unintended consequences of rapidly shifting from external funding to domestic sources warrant careful consideration. While we celebrate the progress made in combating HIV, we must critically point out the challenges of external dependency, limited local influence over fund utilization, and widespread corruption and mismanagement of resources.

Despite its visionary outlook, our agenda confronts substantial implementation hurdles. The diverse political and economic landscapes across African nations raise questions about the uniform applicability of the paper's strategies. Furthermore, integrating public-private partnerships, while crucial, requires navigating complex dynamics between public health objectives and private interests. The position paper's ambition could be grounded further by offering more detailed implementation strategies and establishing robust monitoring and evaluation frameworks. Such measures would provide clearer guidance for overcoming anticipated obstacles and ensure that initiatives are effectively assessed and adapted over time. Furthermore, while the paper's push for community engagement and diverse representation is commendable, these efforts must not be mere token gestures but lead to meaningful participation and decision-making.

For Africa to realize the vision of a sustainable, African-led, and owned HIV response and health agenda, collective action is imperative. African stakeholders must fully support this agenda and claim it as their own in the spirit of Ubuntu. This must be done within the context of continental plans for transformation and revitalization. Together, we can realize the vision of the “Africa we want.”
